# Pharmaceutical pollution: Prediction of environmental concentrations from national wholesales data

**DOI:** 10.12688/openreseurope.14129.2

**Published:** 2022-09-15

**Authors:** Samuel A. Welch, Kristine Olsen, Mohammad Nouri Sharikabad, Knut Erik Tollefsen, Merete Grung, S. Jannicke Moe

**Affiliations:** 1 Norwegian Institute for Water Research, Oslo, NO-0579, Norway; 2 Norwegian Institute of Public Health, Oslo, NO-0213, Norway; 3 Norwegian University of Life Sciences, Ås, NO-1433, Norway

**Keywords:** pharmaceutical pollution, wastewater, wholesale data, predicted environmental concentration

## Abstract

The regulation and monitoring of pharmaceutical pollution in Europe lag behind that of more prominent groups. However, the repurposing of sales data to predict surface water environmental concentrations is a promising supplement to more commonly used market-based risk assessment and measurement approaches. The Norwegian Institute of Public Health (NIPH) has since the 1980s compiled the Drug Wholesale Statistics database - covering all sales of both human and veterinary pharmaceuticals to retailers, pharmacies, and healthcare providers.

To date, most similar works have focused either on a small subset of Active Pharmaceutical Ingredients (APIs) or used only prescription data, often more readily available than wholesale data, but necessarily more limited. By using the NIPH’s product wholesale records, with additional information on API concentrations per product from, we have been able to calculate sales weights per year for almost 900 human and veterinary APIs for the period 2016–2019.

In this paper, we present our methodology for converting the provided NIPH data from a public health to an ecotoxicological resource. From our derived dataset, we have used an equation to calculate Predicted Environmental Concentration per API for inland surface waters, a key component of environmental risk assessment. We further describe our filtering to remove ecotoxicological-exempt and data deficient APIs. Lastly, we provide a limited comparison between our dataset and similar publicly available datasets for a subset of APIs, as a validation of our approach and a demonstration of the added value of wholesale data.

This dataset will provide the best coverage yet of pharmaceutical sales weights for an entire nation. Moreover, our developed routines for processing 2016–2019 data can be expanded to older Norwegian wholesales data (1974–present). Consequently, our work with this dataset can contribute to narrowing the gap between desk-based predictions of exposure from consumption, and empirical but expensive environmental measurement.

## Plain English summary

Pharmaceuticals, by design, affect human or animal biology, targeting specific organs and biological systems to treat diseases. Pharmaceuticals and their metabolites—partly degraded or transformed ingredients—that reach the environment may have unwanted and long-lasting biological effects on plants, animals, and microbes. This comes in addition to environmental footprint of chemicals that are used during the production of pharmaceuticals. In Norway, a coastal nation of more than five million people, the primary route of pharmaceuticals in the environment is via human consumption. Although some pharmaceuticals can be metabolised in the body and degraded in sewage treatment plants, a proportion reaches rivers, lakes, fjords, and coastal zones.

A better overview of the types and amounts of pharmaceuticals in the environment is important for assessing and managing environmental risk, but measuring their presence everywhere can be resource-intensive and expensive. With limited funds for environmental monitoring and management, a rapid and cost-efficient method for predicting concentrations of pharmaceuticals in the environment should be used to screen for the substances most likely to pose a problem.

In this paper we present such an exercise: we worked with the Norwegian Institute for Public Health’s wholesale drugs data, adapting, and translating it from a medical resource to a set of sales weights for each pharmaceutical ingredient. These sales weights were in turn used to predict concentrations of drug pollution in receiving freshwaters. In total, we predicted sales weights and environmental concentrations for almost 900 Active Pharmaceutical Ingredients, from abacavir to zuclopenthixol, sold between 2016 and 2019.

## Introduction

Pharmaceutical consumption is widely recognised as an important source of anthropogenic chemicals in the environment ( European Commission, 2019; Richardson & Bowron, 1985). In much of the European Union (EU) and the European Economic Area, prospective (prior) environmental risk assessments of pharmaceutical products begin with an exposure assessment. Conservative, or worst-case Predicted Environmental Concentrations (PECs) of active pharmaceutical ingredients (APIs) are calculated by extrapolating from the highest average daily dose of a pharmaceutical, and the proportion of a nation’s population taking said pharmaceutical – by default, 1% ( EMA, 2006).

More recently, refined approaches have been suggested using pharmaceutical sales data collected by government agencies or market research agencies, to provide a more accurate and comprehensive prediction of environmental concentrations of APIs at the national ( Grung *et al*., 2008) and European ( Gunnarsson *et al*., 2019) level. In some cases, available data is limited to prescription sales, but where available wholesales data provides a far more complete picture of overall consumption.

In this paper, we present a dataset of predicted API consumption PECs based on reported sales weights of pharmaceuticals from a unique public sector source, the Drug Wholesale Statistics database of the Norwegian Institute for Public Health ( NIPH, 2019). This source covers all sales of pharmaceuticals and medicines to pharmacies, supermarkets, hospitals, and other healthcare providers, from the year 1974 onwards. We describe (1) the sales data and additional information on pharmaceutical API content for the years 2016–2019, (2) the procedures for converting the sales data from number of packets per product to amount (kg) of each API, and (3) a final dataset of total amount of API sold per year, which can be used for prediction of environmental concentration. Although these methods have only been applied to and evaluated for the years 2016–19, they may also be applicable to past data.

With this dataset, we aim to provide an accurate resource describing sales weights and predicted environmental concentrations of environmentally relevant pharmaceutical products sold across Norway, providing a useful snapshot of pharmaceutical pollution for our and others’ work. More advanced modelling approaches, such as ePiE (exposure to Pharmaceuticals in the Environment) ( Oldenkamp *et al.*, 2018), have been developed, but are not yet available for Norway, and though prone to over-estimation our approach permits rapid prioritisation of APIs without the need to gather a great quantity of further excretion and removal data.

In particular, it will provide a useful resource for the characterisation of their environmental risk – on which our work is currently ongoing ( ECORISK 2050 Deliverable D6.2).

## Methods

### Classifications and grouping of pharmaceuticals

**Figure 1.**
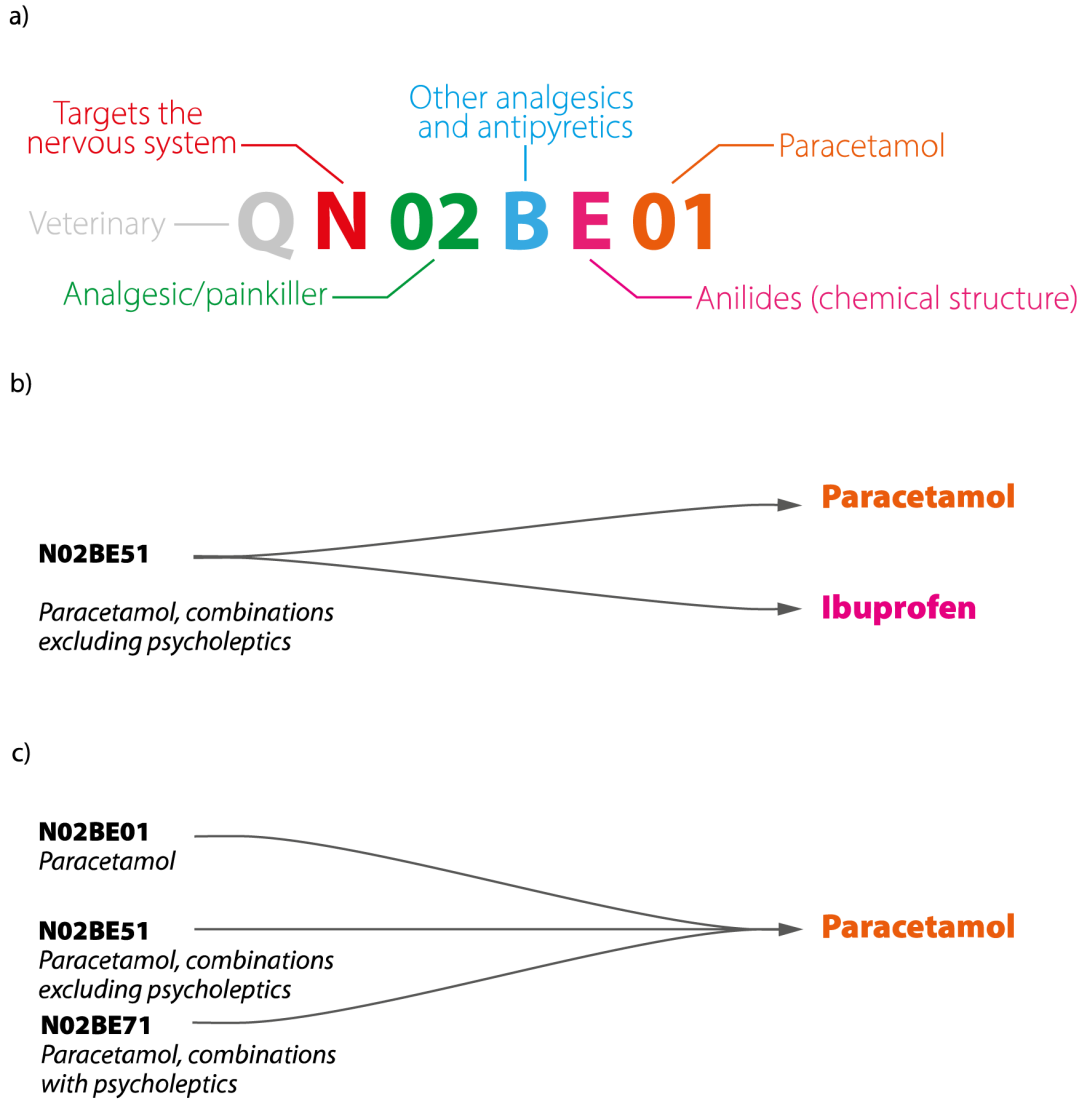
Relationships between APIs and ATC codes. ( ***a***) *An example of the ATC code for paracetamol taken as an analgesic (N02BE01),* ( ***b***) *one ATC code can represent multiple APIs – in this example, N02BE51 represents a combination of paracetamol and ibuprofen, ( **c**) one API can have more than one ATC code, paracetamol is represented here by three codes—N02BE01, N02BE51 and N02BE71—corresponding to the forms and indications it is sold under in Norway. API, Active Pharmaceutical Ingredient; ATC, Anatomical Therapeutic Classification.*

The classification of pharmaceutical substances for human and veterinary use is standardised by the World Health Organization (WHO) under the Anatomical Therapeutic Chemical/Defined Daily Dose (ATC/DDD) code system (RRID:SCR_000677). An ATC code ( Figure 1a) is a seven or eight character tiered alphanumeric code based on the target organ, therapeutic indication and/or pharmacology, and chemical structure of substances, while a DDD is defined as the average maintenance dose for a drug used in its main indication in adults. The ATC system’s widespread global use since the 1970s make it a useful tool for the broad classification of drugs within the Norwegian Drugs Wholesale Database.

ATC codes serve principally as a tool for drug utilization monitoring and research and are difficult to adapt to a substance-driven ecotoxicological approach. APIs are a more relevant entity for the characterisation of environmental risk, as ecotoxicological information is available for individual APIs rather than pharmaceutical products or ATCs. Under the ATC system, a product is characterised by a single ATC code that can contain multiple APIs, which are taken as a cocktail in the same pharmaceutical product ( Figure 1b). Conversely, one API can be used for treatment of diverse disorders of different organs and thereby be associated with different ATC codes ( Figure 1c). This complex set of many-to-many relationships between APIs and ATCs poses a distinct challenge for their interconversion, requiring a great deal of manual cross-referencing of products.

Publications of pharmaceutical sales from WHO Collaborating Centre for Drug Statistics Methodology and the NIPH are given in DDDs, limiting their utility for ecotoxicology work. DDDs aid comparison between pharmaceuticals consumption independent of price, package size and strength, but are impractical for ecotoxicological studies in which the weights of APIs sold are needed and are not always available for individual APIs or combinations of APIs.

Consequently, we elected within our dataset to calculate from scratch overall sales weights for each API, as a proxy of the emission of APIs. This required the assessment of each recorded sold product to determine the mass of each API in the product. The calculation of the total API emission per year is based on (1) the strength of the product ( *i.e.*, the API concentration in units such as mg/L, mg/g, or mg/pill), (2) the amount of the product sold in one package (in units such as L, g, or no. of pills per package) and (3) the number of packages sold per year. See Table 1 for a summary of product and API vocabulary.

**Table 1.** Specific definitions of vocabulary used in this paper.

Vocabulary	Definition
**ATC code**	Anatomical Therapeutic Classification Code, a code classifying APIs or groups of APIs based on their medical use, target human organ, chemical structure, *etc*.
**API**	Active Pharmaceutical Ingredient, the therapeutic chemical(s) in a pharmaceutical product
**Combination drug**	A single product containing more than one API
**Item**	The components of a package, such as individual pills, dispensed sprays of an inhaler, *etc*.
**Package**	A single sold unit of product, such as a packet of multiple sheets of pills, a flask of liquid, *etc*.
**(Pharmaceutical) Product**	A specific manufacturer’s pharmaceutical, as sold, by unique product ID
**Strength**	The amount of a given API in an Item, Package or Product
**Unit**	The unit assigned to a given Strength, such as mg L ^-1^, mg pill ^-1^, International Units, *etc*.
**DDD**	Defined Daily Dose, “the average maintenance dose per day for a drug used in its main indication in adults” ( WHOCC, 2018), a standardised unit per ATC code and route of administration used to give a rough estimate of consumption.

### Active Pharmaceutical Ingredients

Most—more than 50% in 2007—APIs are sold as pharmaceutical salts, with positive or negatively charged ions appended to their structures to increase stability and solubility in water ( Bastin *et al*., 2000; Paulekuhn *et al*., 2007). Where the given mass of API in a product in fact refers to the salt form, this can lead to over-estimation of the total volume of active substance sold, especially where the ion represents a substantial portion of the overall weight. Information on the salts used in each product was not always listed in the source data, and consequently, we assumed the full given mass of API per product referred to the active ion However, we aim to include an assessment of the effects of salts on PECs in future analyses of the data.

### Data sources and management

**Figure 2.**
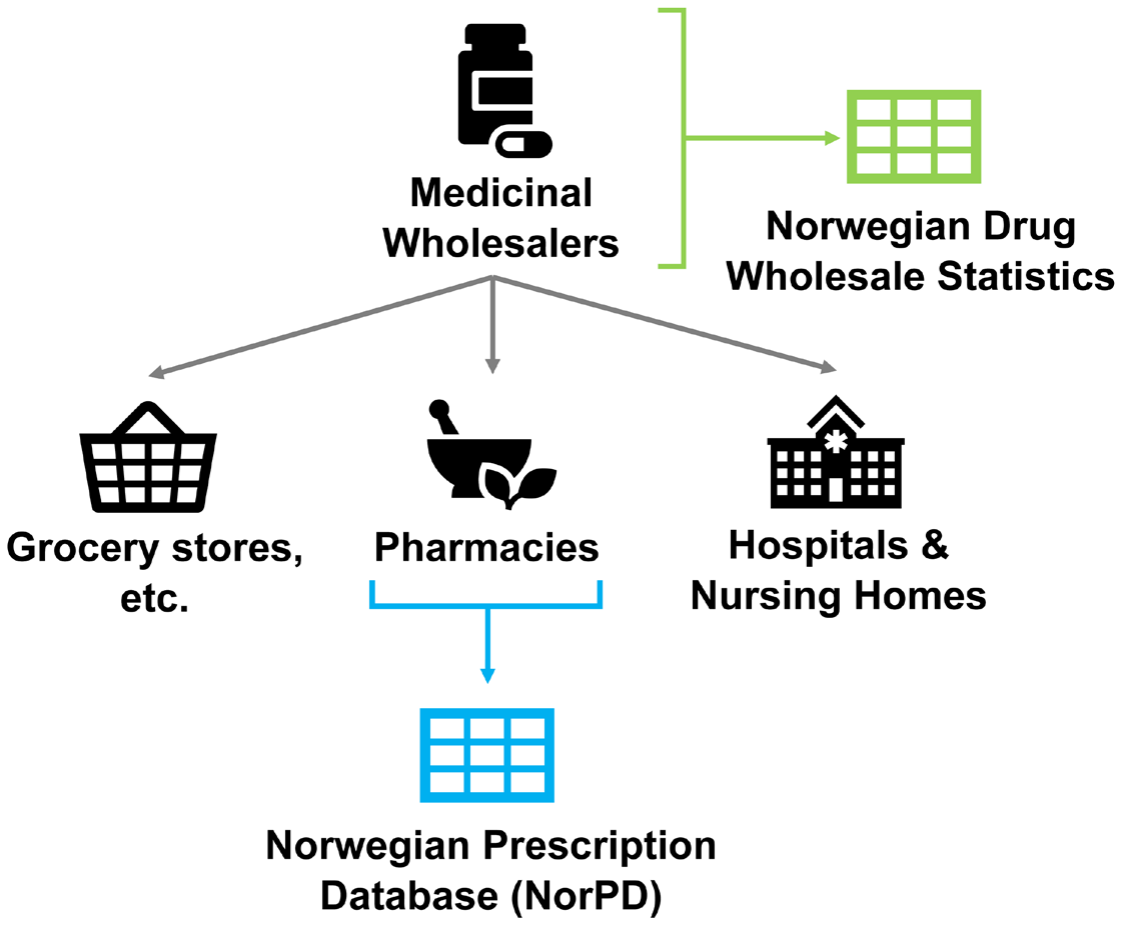
Diagram of information sources to NIPH Norwegian Drug Wholesale Statistics and Norwegian Prescription Database. *Figure reproduced and adapted from* *Sommerschild et al. (2021a)* *with permission from the publisher. The Norwegian Prescription Database is, at time of writing, in the process of being renamed to the Norwegian Prescribed Drug Registry.*

**Figure 3.**
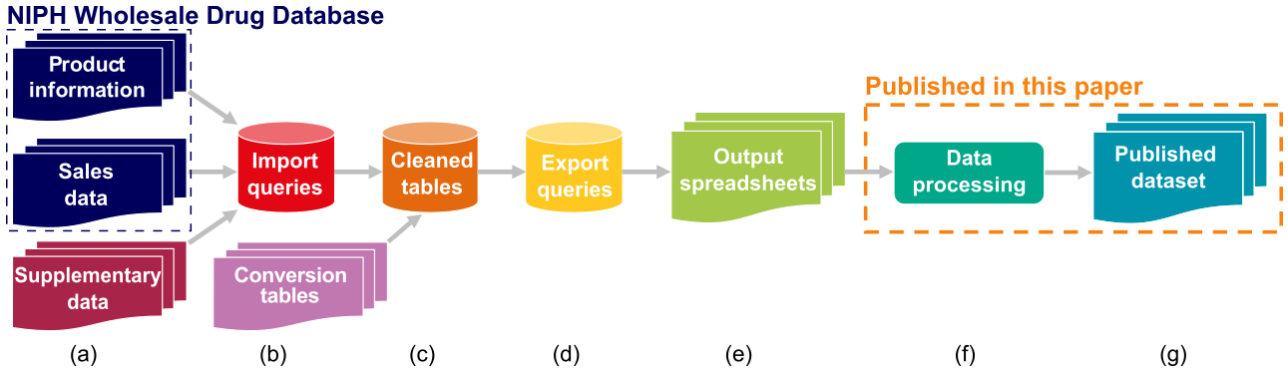
Simplified diagram of data extraction and management pipeline. Sales and product background data ( **a**) from NIPH (dashed blue box) and elsewhere was imported into an Access DB via a series of queries ( **b**), cleaned with the addition of various conversion tables ( **c**), and exported ( **d**) into output spreadsheets ( **e**). This data was then formatted for analysis in R ( **f**) and PECs calculated, and the results output to foreground CSV files ( **g**), both of which are available as part of this paper. NIPH, Norwegian Institute of Public Health.

Sales data for years 2016–2019 were extracted from the Norwegian Drugs Wholesale Database ( Figure 2, Figure 3a, Sales data). By contrast to prescription-only records such as NorPD (Norwegian Prescription Database) this covers all sales to pharmacies, hospitals, nursing homes, and non-pharmacy outlets licensed to sell drugs within Norway, including prescriptions, over-the-counter (OTC) sales, and procurement by medical establishments ( NIPH, 2019). In its raw form this dataset consisted of per-product sales, such as a packet containing multiple sheets of pills, or a suspension of liquid medicine.

The Norwegian health system distinguishes between three groups of human prescription medications. Group A and B cover drugs with potential for abuse, such as stimulants, opiates and strong painkillers, while Group C includes drugs minimal potential for abuse but that are still controlled, such as anti-depressants. All other products are available OTC. For the purposes of this analysis, Groups A, B and C were combined. Note that in some cases, an API may be available both on prescription and OTC – for instance, smaller doses of paracetamol can be bought OTC, while larger doses require a prescription ( Helsenorge, 2020). The Norwegian Drug Wholesale Statistics and its output “wholesale data” covers both prescription and OTC sales of human and veterinary medications.

In adherence with NIPH’s commercial confidentiality requirements, sales in currency values, and commercially sensitive information on the sales of individual manufacturers’ products were removed from the final published dataset.

Additional information on individual products that was required for calculating the sales weight per API ( Figure 3a, Product information), including number of items per package, strength (concentration of API per item), and associated unit were obtained separately from the centralised NIPH sales database and matched to sales data using internal product codes. In a sizable number of cases, no additional data were available for given products, automatic matching failed, or the data available were inappropriate for use in our workflow. Here records were checked manually against product contents records online, principally the Norwegian pharmaceuticals specialties site Felleskatalogen, the UK Electronic Medicines Compendium, and the US site Drugs.com. Cases where one product contained two or more APIs (combination drugs) were split into separate entries for each API to ensure substances were fully accounted for.

Although efforts were made to include the sales of as many products as possible, products with sales below 1000 packages over the four-year period, except for categories of special interest (antibiotics, sex hormones), were excluded as a time-saving measure. Additionally, gas APIs (such as anaesthetic gases) were likewise excluded.

**Figure 4.**
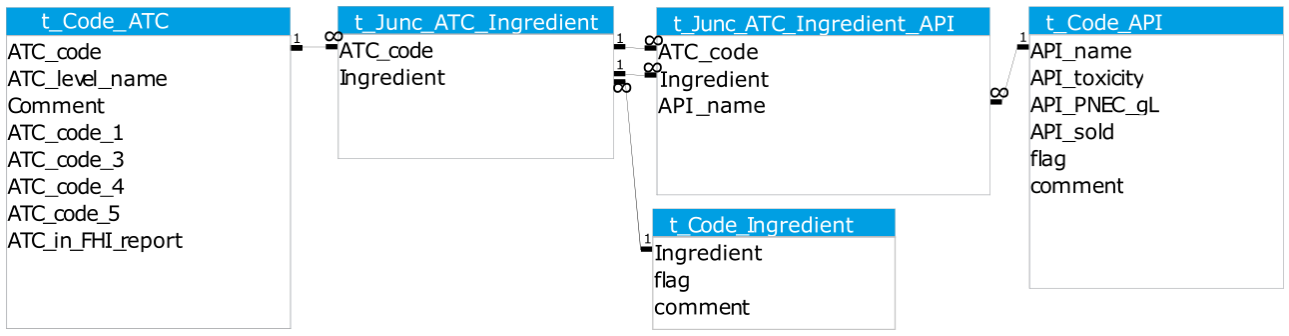
Simplified diagram of database structure: the main data tables. API, Active Pharmaceutical Ingredient; ATC, Anatomical Therapeutic Classification; PNEC, Predicted No-Effect Concentration.

The two primary data sources, and supplementary product information where gaps were present in the former, were imported into a Microsoft Access database and organised into a related set of tables. The main table types were data tables, conversion tables, and code lists. The main data tables are shown in Figure 4 and described below.

1) t_Product: the description of each pharmaceutical product (identified by product number), including information on the product type and the product amount per package ( Table 2)
2) t_Product_API: the concentration of each API per item and the total amount of API per package of the product ( Table 3)
3) t_Sales_Product: the number of packages sold per product per year ( Table 4)

**Table 2.** Field names, types, and descriptions for the Product Table t_Product.

Field name	Data type	Description
**ProductCode**	Number	Database internal unique product ID
**ProductName**	Short Text	Full product name from NIPH records
**ProductName_short**	Short Text	Product name with medium/dose removed
**ATC_code**	Short Text	Full ATC Code
**ProductDetails**	Short Text	Additional medium/dose data from ProductName
**ProductType**	Short Text	Standardised medium: pill, fluid, *etc*.
**ProductGroup**	Number	Formulation institution; 1 for manufacture, 2 for compounding pharmacy
**DateStart**	Date	Unused variable for product registration date
**DateEnd**	Date	Unused variable for product removal date
**PackageQuantityValue**	Number	Quantity of medium per package (number of pills, L of fluid, *etc*.)
**PackageQuantityUnit**	Short Text	Unit of medium per package
**Item**	Short Text	Unused variable from source data
**ConversionFactor**	Number	Unused variable from source data
**NoOfAPI_PerProduct**	Number	Number of APIs in a product
**NoOfItemsPerPackage**	Number	Unused variable superseded by PackageQuantityValue

NIPH, Norwegian Institute of Public Health; ATC, Anatomical Therapeutic Classification; API, Active Pharmaceutical Ingredient.

**Table 3.** Field names, types, and descriptions from the API per Product Table t_Product_API.

Field Name	Data Type	Description
**ProductCode**	Number	Database internal unique product ID
**API_name**	Short Text	
**StrengthValue**	Number	Original strength information from NIPH (not standardised)
**StrengthUnit**	Short Text	Original strength information from NIPH (not standardised)
**API_ConcentrationPerItemValue**	Number	Converted API strength value (with standardised unit if possible)
**API_ConcentrationPerItemUnit**	Short Text	Standardised API strength unit (if possible)
**API_AmountPerPackageValue**	Number	Calculated API amount value (with standardised unit if possible)
**API_AmountPerPackageUnit**	Short Text	Standardised API amount unit (if possible)
**Comment**	Short Text	
**Exclude**	Short Text	Yes (if record should be excluded from extraction)

NIPH, Norwegian Institute of Public Health; API, Active Pharmaceutical Ingredient.

**Table 4.** Field names, types, and descriptions from the Product Sales Table t_Sales_Product.

Field Name	Data Type	Description
**sYear**	Number	Sales year
**ProductCode**	Number	Database internal unique product ID
**PackageAmountSoldValue **	Number	Number of packages of a unique product sold
**PackageAmountSoldUnit **	Short Text	Helper variable used to record counting process

Information on APIs in a given product was not available in the original data sources but had to be extracted from the ATC codes associated with the sales data ( Figure 3a). In some cases, extracted data corresponded directly to an API, but for combination products, and ATC codes where the included APIs were not immediately interpretable, API content was determined, stored, and converted at the individual product level. Ultimately, for each product ( Table 2), the associated API names were extracted from the full ATC name and entered in the table t_Product_API ( Table 3).

In most cases the information needed for calculating the amount of API per package (the concentration of API in the product and the amount of the product per package) was available in the original data source (the product information table). In some cases, where this information was not provided, it was still possible to extract the information manually from the product name.

For products where API information could not be found in the included data, it was instead sourced for each individual product from the Norwegian pharmaceutical specialties website Felleskatalogen or Summaries of Product Characteristics (SPCs) from the pharmaceutical specialties websites of other nations ( Electronic Medicines Compendium (UK), Pharmaceutical Specialties in Sweden, Medical Online Information Centre (Spain)). This was also the case for combination products containing two or more APIs, which typically required further work to determine and confirm the APIs present.

**Figure 5.**
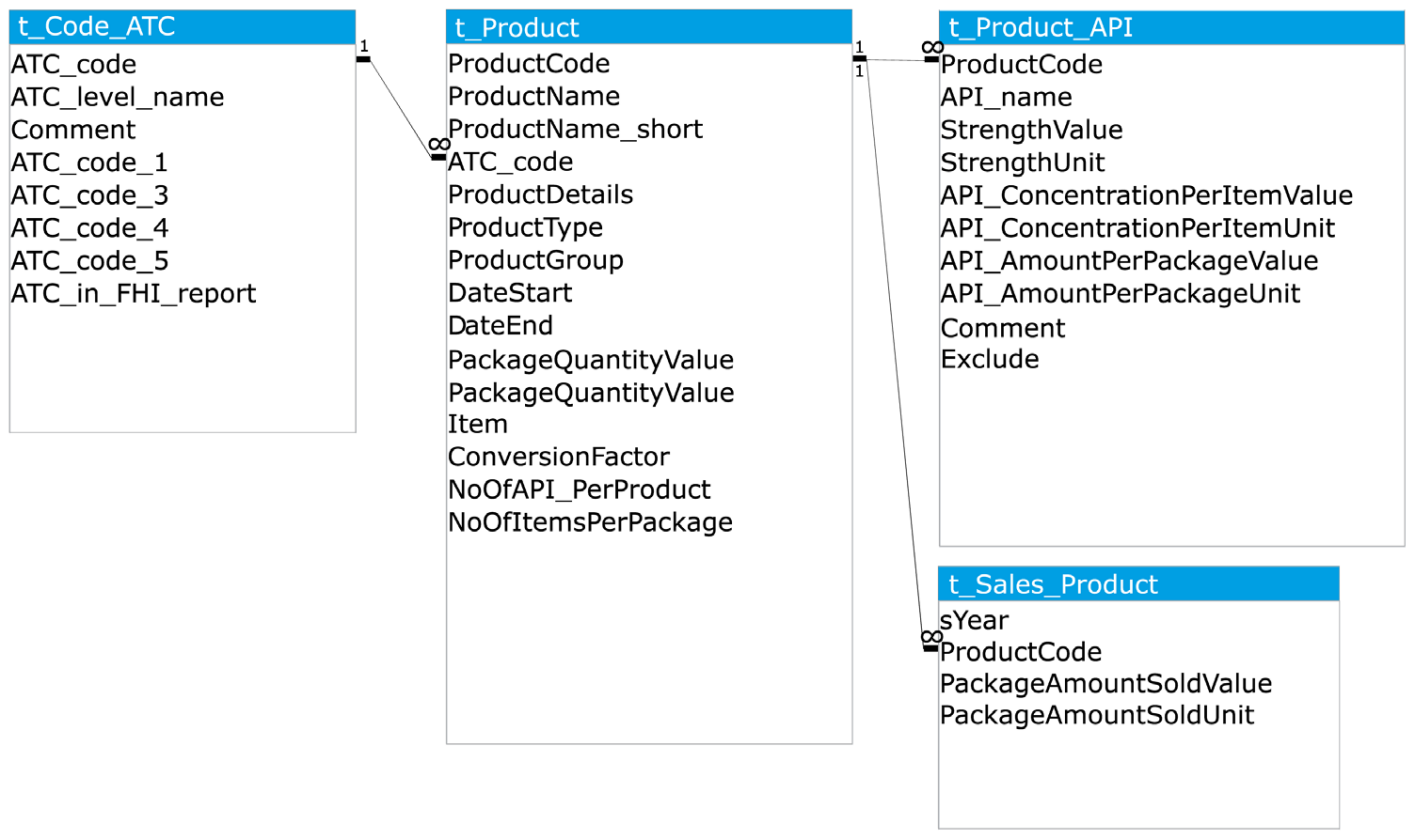
Diagram of code lists and conversion tables. Defines the many-to-many relationships between ATC and API in database. ATC, Anatomical Therapeutic Classification; API, Active Pharmaceutical Ingredient.

The resulting many-to-many relationship between ATC and API (see Figure 1) is represented by the code lists and junction tables shown in Figure 5.

Finally, the information on yearly sales (number of packets) per product was stored in the table t_Sales_Product ( Table 4). During data extraction ( Figure 3d), this yearly sales information was combined with the calculated amount of API per product package, to obtain the total amount of API per year from the sales data.

### Data processing in R

Data extracted from the Access database ( Figure 3d) were subsequently exported into flat files ( Figure 3e) for calculation of PECs and future analysis. For this purpose, the records were grouped by API and year and the calculated amount sold aggregated by sum. The exported dataset was prepared for analysis and publication in R version 4.1.2 “ *Bird Hippie*” ( R Core Team, 2021; RRID:SCR_001905). A full list of the R packages used is available as *Underlying data* ( Welch *et al*., 2022).

**Figure 6.**
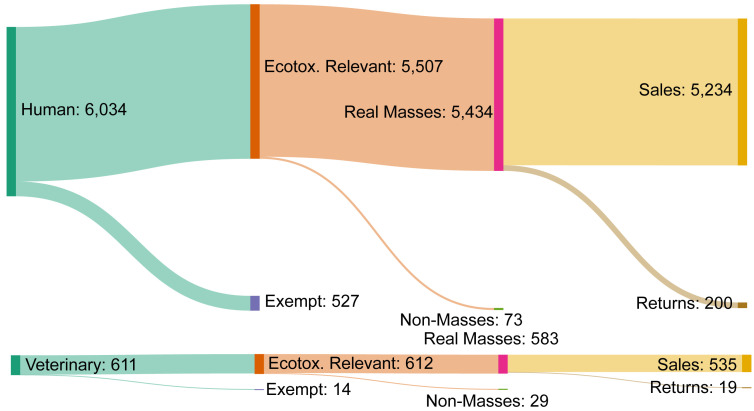
Records retained/removed at each stage of data processing. Count of unique products sold in 2019 retained and removed at each step of data processing ( Figure 3f), categorised as human (upper) or veterinary (lower). Stages cover the removal of exempt product types (vaccines, vitamins, etc.), substances with sales recorded in non-mass units (e.g. international units), and negative sales corresponding to the return and disposal of products.

Sales weights per product per year were filtered to remove any zero values, and values for which no units were assigned, representing records for which the API amount could not be calculated. Sales weights were then summed by API, per year, and APIs were filtered according to a list of exemptions from risk assessment on the basis of non-toxicity (as applies to vitamins, vaccines, antibodies, *etc*. ( EMA, 2006)). Unique products excluded at each state are illustrated in Figure 6, and the total number of entries input (unique products) and APIs output are summarised in Table 5. The final dataset is published as a comma-separated values (.csv) file.

**Table 5.** Table of number of unique human and veterinary products input from starting dataset ( Figure 3e) and number of unique API output ( Figure 3g), by year.

	Starting dataset entries		Unique APIs
**Year**	*Human*	*Veterinary*	
**2016**	5,713	660	804
**2017**	5,904	655	820
**2018**	5,991	611	820
**2019**	6,034	597	831

API, Active Pharmaceutical Ingredient.

***Graphics.*** Graphs were rendered in R (see repository for code and packages used ( Welch *et al*., 2022)). Diagrams were drawn in Adobe Illustrator (RRID:SCR_010279), with the exception of Figure 6, which was rendered by the website SankeyMATIC.

### Data evaluation

The predicted sales weights in this dataset were compared to similar datasets gathered by both co-authors in NIPH and other Norwegian agencies ( Table 6) in order to detect discrepancies and assess the correspondence between independently calculated PECs. Although the primary output of this data paper is PECs, their limited availability made it more practical to carry out comparisons at the sales weights level, particularly as the choice of variables in the calculation of PECs is a question of judgement and conservatism as well as mathematics.

**Table 6.** Summary and labelling scheme for datasets used and referenced in this paper.

Label	Source	Type	Output format	Years used (Total coverage)	Reference
**Welch**	NIPH	Wholesale	g/API	2016–19	DOI: https://doi.org/10.17605/OSF.IO/GMX58
**Felleskatalogen**	FK	Wholesale	g/API	2018	Felleskatalogen, 2022
**NorPD**	NIPH	Prescription	DDDs	2016–19 (2004–20)	NIPH, 2021
**Grung**	NIPH	Wholesale	DDDs & g/API	2005	Grung *et al.*, 2008
**NIPH**	NIPH	Wholesale	DDDs	2007–19	Sakshaug *et al.*, 2013; Sakshaug *et al.*, 2018; Sommerschild *et al.*, 2021b

NIPH, Norwegian Institute of Public Health; NorPD, The Norwegian Prescription Database; API, Active Pharmaceutical Ingredient; DDD, Defined Daily Dose.

The choice of datasets for comparison and data evaluation was informed mainly by the scarcity of publicly available data in Norway, compared to better studied nations such as Germany or Spain. The Grung dataset was chosen for comparison as the only previously published dataset using the same method.

The Norwegian Pharmaceutical Specialties website Felleskatalogen maintains a rolling risk assessment on a yearly basis of pharmaceutical risk, using sales data from a private market research firm. In order to benchmark the completeness and accuracy of our dataset to another party’s measurement of the same values, we compared our calculated sales weights to theirs. Due to the data’s private ownership, Felleskatalogen’s PECs are not archived year-on-year or especially transparent; this makes them a useful resource for comparison, but not a permanent part of the scientific record.

Comparisons were performed using a Bland-Altman plot, also known as a Tukey mean-difference plot ( Bland & Altman, 1999), which allows for the visual comparison of two measurements of a single parameter.

Further comparisons were conducted between our dataset and prescription data for a high-use subset of APIs. NorPD is a publicly available resource, comparable to those available in other nations, that can produce reports of drug consumption by age, region, sex, and year across Norway. However, as a record of prescription this database is necessarily more limited than the Drug Wholesale Statistics database; additionally, all sales are recorded only in DDDs, introducing inaccuracy compared to actual quantities sold, and excluding drug formulations for which no DDD has been assigned. A further Tukey mean-difference plot (also known as a Bland-Altman)plot was created to compare prescription and wholesales predicted sales weights.

Lastly, we compared our predicted sales weights to two further analyses based on the same dataset. An analysis of 2005 API sales weights for a panel of 11 APIs was conducted by Grung *et al*. (2008); we selected three high-use APIs with a wide range of constituent ATC codes—paracetamol, ethinylestradiol and ibuprofen—and compared these sales weights with our predictions for 2016–19.

To further benchmark trends in consumption, these sales weights were normalised by dividing the figures by the annual population of Norway. They were then compared to wholesale data published by NIPH – available as PDF reports ( Sakshaug *et al*., 2013; Sakshaug *et al*., 2018; Sommerschild *et al*., 2021b) of consumption in DDDs per thousand people per day for a limited range of substances. Although direct comparisons between normalised sales weights and DDD/1000 people/day were not possible, we were able to compare overall trends in consumption to look for disagreement.

### Predicted Environmental Concentrations

PECs of individual APIs in the compartment Surface Water were calculated using a modified form ( Equation 1) of the standard refined PEC _SW_ equation, with default variables ( Table 7), outlined in the EMA’s guidelines for pharmaceutical environmental risk assessment (2006).

**Table 7.** Table of PEC _SW_ equation default variables and parameters.

Component	Unit	Description
**g of API sold**	*g year ^-1^*	The total weight (g) of an API sold in a year
**WWTP removal**	*unitless*	The proportion of the API removed at WWTP (default of 0)
**365**	*days year ^-1^*	The number of days in a year
**Wastewater consumption**	*L person ^-1^ day ^-1^*	The average wastewater consumption (L) of the population of a given area per day
**Population**	*persons*	The population of a given area
**Dilution factor**	*unitless*	The ratio of dilution between WWTP effluent and receiving waters (default of 10)

PEC, Predicted Environmental Concentrations; API, Active Pharmaceutical Ingredient; WWTP, Wastewater Treatment Plant.

As no specific bodies of water are specified in the guidelines, the model is assumed to apply to all relevant freshwater bodies, *i.e.*, rivers and lakes. In Norway, where a significant proportion of WWTP (Wastewater Treatment Plant) outflow is to saltwater fjords, the omission of marine modelling is a limiting factor, but is in-line with current practice in Norway.

Likewise, metabolism of APIs in the human body was assumed to be 0 as a worst-case scenario for all APIs. Although this may overestimate PECs, the assumption that metabolism of an API intrinsically removes the overall volume of ecotoxicologically active substance entering the environment may also underestimate the effects of metabolites ( Farré *et al.*, 2008).

Equation 1.


$$PEC_{SW}=\frac{API\;sold\;\times \;(1-WWTP\;Removal)}{365\;\times \;Wastewater\;consumption\;\times \;Population\;\times \;Dilution\;factor}$$

PEC, Predicted Environmental Concentrations; API, Active Pharmaceutical Ingredient; WWTP, Wastewater Treatment Plant.

As mentioned, the standard equation estimates sales weights from the maximum dose of a given API and the proportion of people in a population taking that API. By contrast, by using our dataset of pharmaceutical wholesales we can input a more exact figure for consumption across the entire population of Norway. Default values for removal in wastewater treatment plants (0% removal) and dilution factors (dilution to 1 part in 10 upon entering receiving waters) were retained as worst-case assumptions, potentially contributing to overestimation of PECs. In particular, the assumption of 0% removal biases the dataset towards overestimating concentrations of well-removed APIs.

In addition, the default dilution factor of 10 has been criticised as potentially not covering especially low-flow conditions in European rivers ( Link *et al.*, 2017). In Norway, the coast and sea are the primary receivers of Norwegian treated wastewater ( Berge & Sæther, 2020); information on dilution factors is difficult to locate, but one report ( Källqvist *et al.*, 2002) suggested coastal WWTP outflow pipes are situated at sufficient depth and distance to achieve dilution rates of 50–75.

PECs were individually calculated per API, per year, using information on yearly average wastewater generation and Norwegian population, obtained from Statistics Norway and included as *Underlying data* ( Welch *et al*., 2022).

### Identification and grouping of APIs

To aid in the contextualisation and machine reading of the dataset, additional data were collected and appended to API sales data. Firstly, standard InChIKeys, a short, unique string based on molecular structure, were, where possible, found for all APIs ( Heller *et al*., 2015) using the R package webchem ( Szöcs *et al*., 2020) (RRID:SCR_017684) to look up API names via the Chemical Translation Service ( Wohlgemuth *et al*., 2010) (RRID:SCR_014681).

Additionally, APIs were sorted into single categories based on function and/or target organ (antidepressant, respiratory, antibacterial, *etc*.), adapted from ATC classifications and sourced from Felleskatalogen, Drugs.com, and WHOCC for Drug Statistics records. A short description of the type and application of APIs was also included, based principally but not exclusively on use in Norway.

## Data evaluation

### Comparison with Felleskatalogen data

**Figure 7.**
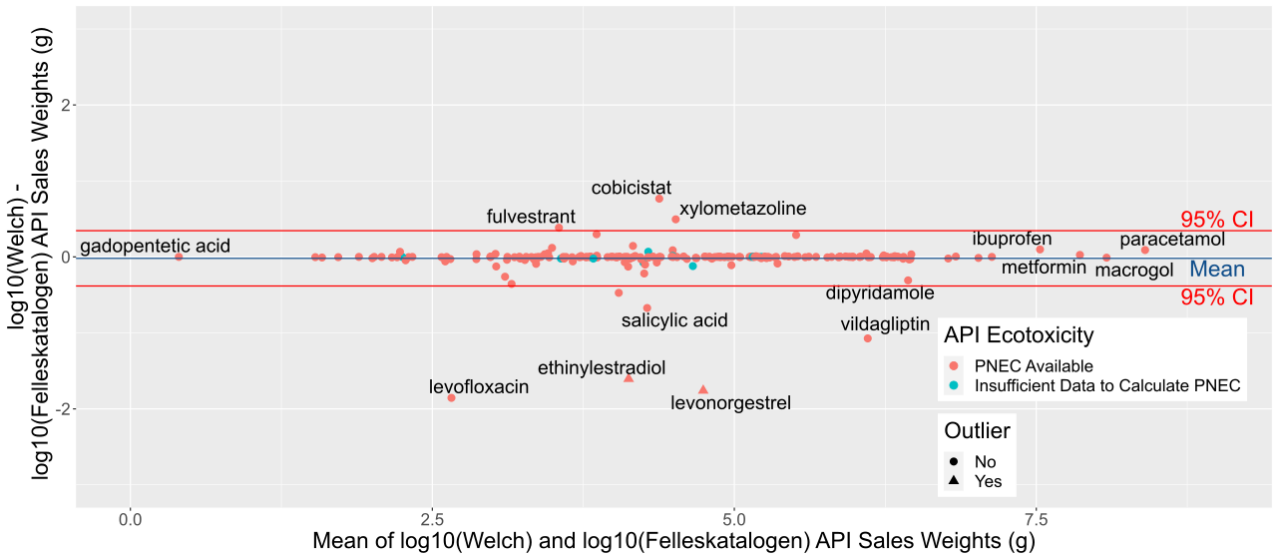
Comparison between NIPH-derived and Felleskatalogen Predicted Environmental Concentrations datasets, for sales in 2018. Bland-Altman or Tukey mean-difference plot of difference (y axis) and mean (x axis) of log10-transformed sales weight data from our and Felleskatalogen sources. Blue line marks mean difference, and red 95% Confidence Intervals. A substance with no difference between the two predicted weights would fall on the 0 line on the y axis. NIPH, Norwegian Institute of Public Health; API, Active Pharmaceutical Ingredient; PNEC, Predicted No-Effect Concentration.

Figure 7 summarises agreement between the two datasets for the year 2018. A mean difference (blue line) extremely close to zero on the y-axis indicates little average difference between calculations. However, a number of substances below the lower red line (95% CI) indicate potential errors in either our or Felleskatalogen’s calculations ( Table 6). In total, Felleskatalogen sales weights are available for 203 APIs, of which 193 have available toxicity data in the form of Predicted No Effect Concentrations (PNECs), while our dataset contains sales weights for 821 APIs, 255 of which have available PNECs.

Of these, discrepancies between figures for ethinylestradiol and levonorgestrel are due to the mistaken substitution of milligrams (mg) for micrograms (mcg or μg) for one combination product containing levonorgestrel and ethinylestradiol in Felleskatalogen’s data source and have consequently been excluded from summary statistics. Differences in sales of salicylic acid may be due to its presence in a number of non-medical skin products not included in NIPH data, and/or from the combination of the weights of salicylic acid and 5-aminosalicylic acid, treated as separate APIs in our data. The discrepancy for levofloxacin between our data (5.4 × 10 g) and Felleskatalogen (3.9 × 10 ^3^ g) is likely due to the exclusion of eye drops containing the antibiotic from the NIPH source data, while no explanation was found for the difference in vildagliptin, 3.7 × 10 ^4^ g compared to 4.4 × 10 ^6^ g.

### Comparison with prescription data

**Figure 8.**
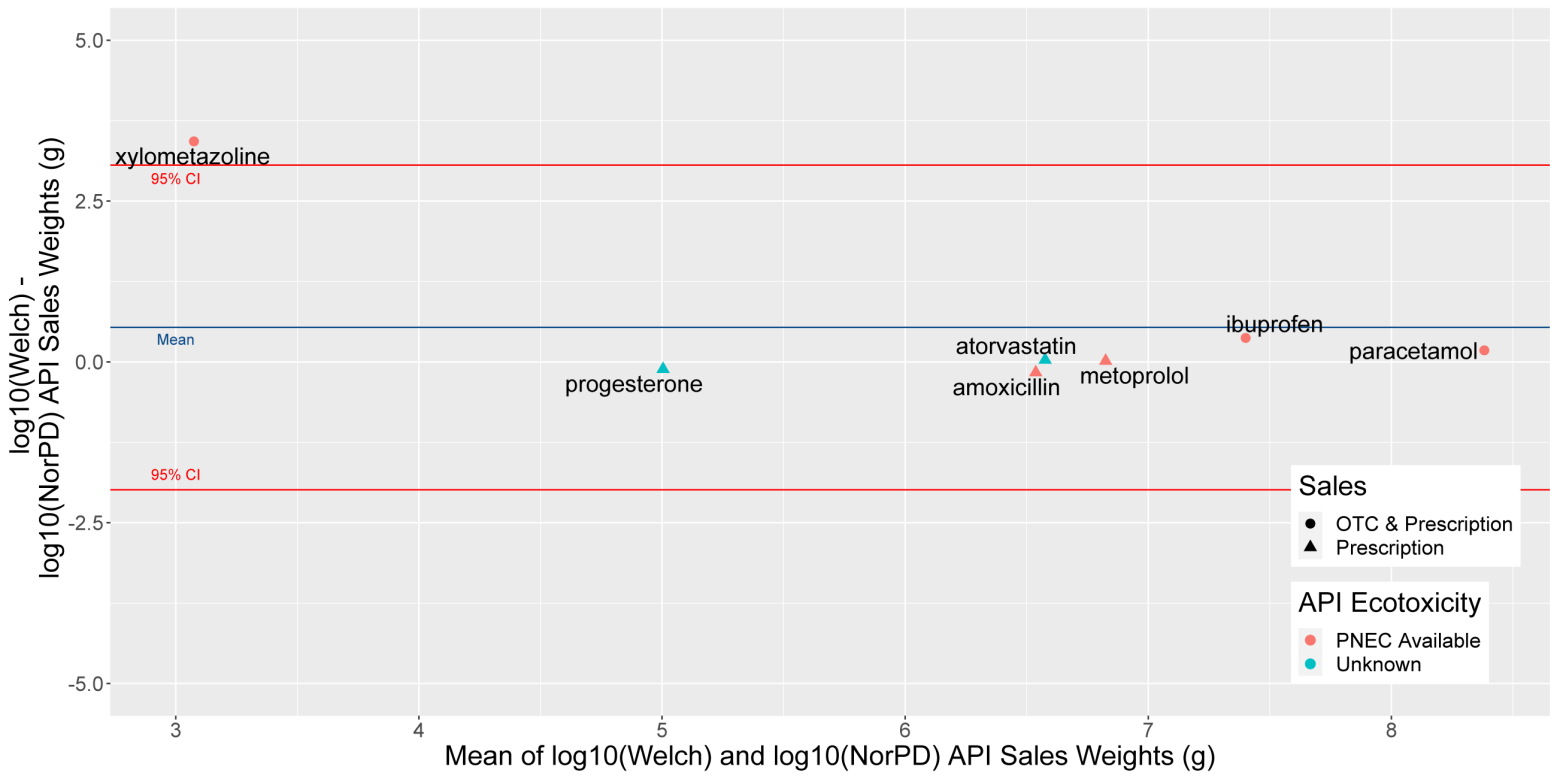
Bland-Altman or Tukey mean-difference plot of difference (y axis) and mean (x axis) of log10-transformed sales weight data from our and NorPD sources for six selected APIs in 2019. Blue line marks mean difference, and red 95% Confidence Intervals. A substance with no difference between the two predicted weights would fall on the 0 line at the centre of the y axis. NorPD, The Norwegian Prescription Database; API, Active Pharmaceutical Ingredient; OTC, over the counter; PNEC, Predicted No-Effect Concentration.

To assess the value of our dataset compared to NorPD ( Table 6), we compared predicted sale weights for six substances ( Table 8) present in both datasets, a selection of common human, veterinary, over the counter (OTC) and prescription APIs, for the year 2019 ( Figure 8).

**Table 8.** Panel of human and veterinary drugs selected for comparison between our dataset and NorPD. Where multiple DDD values were possible for one ATC code, the highest value was used. Codes beginning with Q correspond to veterinary applications. Inj. refers to injected forms of drug, vag. to vaginal.

API	Description	Availability	ATC Codes	DDD	Notes
**Paracetamol**	Human painkiller	OTC & Prescription	N02AJ06 N02BE01 N02BE51	3.0 g (oral) 3.0 g (oral) 3.0 g (oral)	High consumption
**Ibuprofen**	Human painkiller	OTC & Prescription	M02AA13 C01EB16 M01AE01	N/A 0.03 g (oral) 1.2 g (oral)	High consumption
**Xylometazoline**	Human nasal decongestant	OTC & Prescription	R01AA07 R01AB06	0.8 mg (nasal) N/A	High consumption
**Amoxicillin**	Human & vet. antibacterial	Prescription	J01CA04 J01CR02 QJ01CA04	1.5 g (oral) 3 g (inj.) 1.5 g (oral) 3 g (inj.) N/A	Significant consumption
**Progesterone**	Human & vet. sex hormone	Prescription	G03DA04 QG03DA04	30 mg (oral) 5 mg (inj.) 90 mg (vag.) N/A	High consumption
**Atorvastatin**	Human statin	Prescription	C10AA05 C10BA05	20 mg (oral) N/A	2 ^nd^ most used prescription
**Metoprolol**	Human beta blocker	Prescription	C07AB02	0.15 g (oral)	9 ^th^ most used prescription

NorPD, The Norwegian Prescription Database; API, Active Pharmaceutical Ingredient; DDD, Defined Daily Dose; ATC, Anatomical Therapeutic Classification; OTC, over the counter; N/A, not applicable.

Comparing wholesale and prescription sales weights for these substances ( Table 8), it can be seen that on average, prescription data predicted lower sales weights for APIs, but this was driven by the decongestant xylometazoline, whose sales weight was predicted to be around 1000 times higher than prescription weight. The OTC and prescription painkillers paracetamol and ibuprofen had a sales weight of roughly 1.5 times and 2.3 times wholesale than prescription.

The prescription-only APIs metoprolol and atorvastatin showed strong agreement between wholesale and prescription weights (<10% difference), while amoxicillin and progesterone were predicted a 45% and 28% higher prescription weight than sales weight. In both cases, this is likely due to the difficulty of distinguishing the appropriate DDD to use with prescription data, as it does not distinguish between routes of admission at the ATC code level, and the highest DDDs for these substances are 2–3 times higher than the lowest.

### Comparison with Grung *et al*., 2008 and NIPH Wholesale Report Data

**Figure 9.**
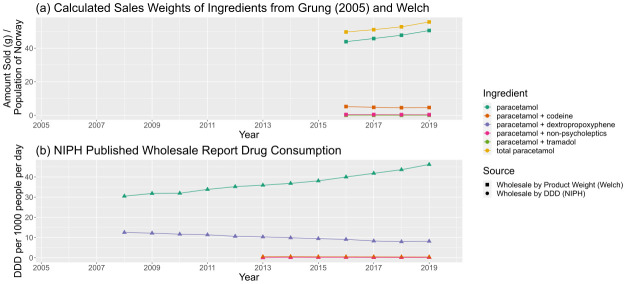
Comparison of predicted sales data sources for paracetamol and paracetamol-containing products. ( **a**) Calculated sales weights, by ingredient, for products containing paracetamol in 2005 and from 2016–19, normalised by annual population of Norway. ( **b**) Consumption of paracetamol-containing products by ingredient from NIPH published reports, in DDD per 1000 people per day. The combination “paracetamol + non-psycholeptics” corresponds to combinations of paracetamol with caffeine, acetylsalicylic acid, or ibuprofen. For a more complete description of data sources, refer to Table 6. NIPH, Norwegian Institute of Public Health; DDD, Defined Daily Dose.

Predicted sales weights, normalised by population, were also compared to earlier (recorded in 2005, published in 2008) ( Table 6) predictions and (non-comprehensive) published trends in consumption by DDD. Comparing our predictions of paracetamol sales weights to those in 2005 ( Figure 9) shows a plausible growth in normalised consumption, the majority of which is driven by growing consumption in plain paracetamol over time.

**Figure 10.**
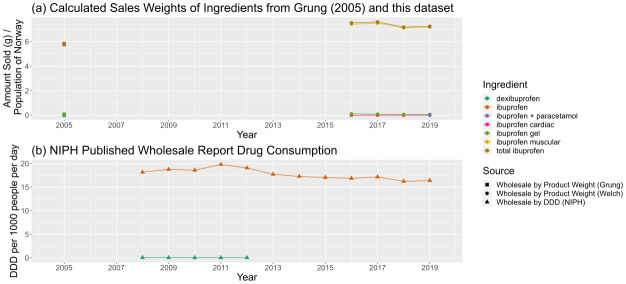
Comparison of predicted sales data sources for ibuprofen and ibuprofen-containing products. ( **a**) Calculated sales weights, by ingredient, for products containing ibuprofen in 2005 and from 2016–19, normalised by annual population of Norway. ( **b**) Consumption of ibuprofen-containing products by ingredient from NIPH published reports, in DDD per 1000 people per day. For a more complete description of data sources, refer to Table 6. NIPH, Norwegian Institute of Public Health; DDD, Defined Daily Dose.

Consumption of ibuprofen ( Figure 10) is also driven by the consumption of ibuprofen as a painkiller (variously classified as M01AE01 (oral/rectal/injected) and M02AA13 (topical)). Drawing direct comparisons between different combinations of the API is difficult due to changes in API encoding, patchy data availability in Wholesale Reports, and the disappearance of dexibuprofen, an enantiomer of ibuprofen. Nevertheless, in overall trends, a similar pattern of overall decline offset by a small bump in 2017 can be observed.

**Figure 11.**
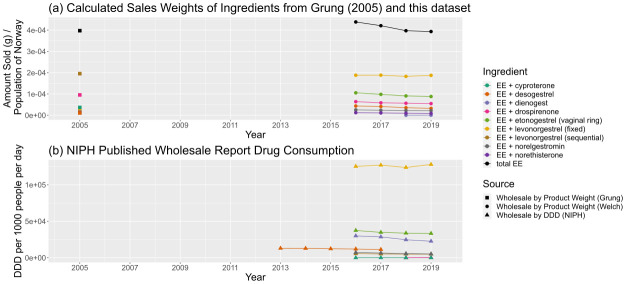
Comparison of predicted sales data sources for ethinylestradiol and ethinylestradiol-containing products. ( **a**) Calculated sales weights, by ingredient, for products containing EE in 2005 and from 2016–19, normalised by annual population of Norway. ( **b**) Consumption of EE-containing products by ingredient from NIPH published reports, in DDD per 1000 people per day. Fixed and sequential ingredients refer to a course of pills of either a fixed dose, or a changing (sequential) dose. For a more complete description of data sources, refer to Table 6. NIPH, Norwegian Institute of Public Health; DDD, Defined Daily Dose; EE, ethinylestradiol.

Interpreting individual sales patterns for ethinylestradiol, also known as EE, is harder than the above due to the wide range of combination contraceptives and hormone therapies. An overall trend of decline in consumption in Figure 11a can be seen, driven by small decreases in constituent consumption, but in Figure 11b it is less apparent whether the trends of different compositions balance each other out. Historical data on ethinylestradiol consumption was largely absent in Wholesale Reports before 2016 ( Sakshaug *et al*., 2013; Sakshaug *et al*., 2018), except in the case of vaginal rings, where consumption was given in units sold in one report and DDD in the next, making comparisons difficult. Nevertheless, trends for individual combinations that appear in both datasets – EE and levonorgestrel (in fixed static doses), vaginal rings containing EE and etonogestrel, and EE and cyproterone showing corresponding trends.

### Checking for extreme changes

**Figure 12.**
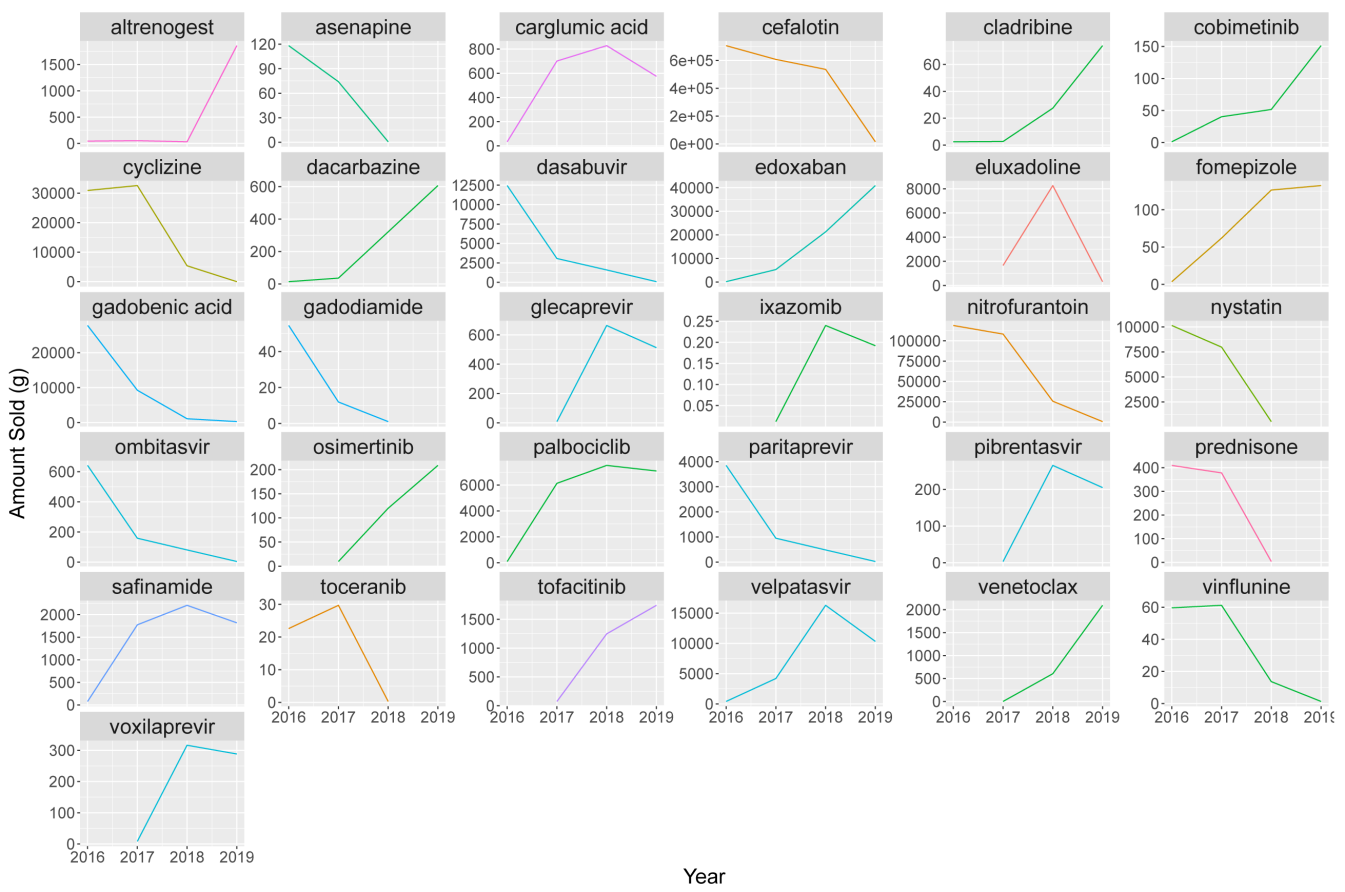
Calculated sales weights 2016–2019 for APIs where at least one year’s weight is 10x bigger or smaller than the mean API sales weight. A total of 31 APIs were shortlisted under this criterion; see Table 9 for further details. Coloured by type. API, Active Pharmaceutical Ingredient.

In addition to the above comparisons of our data with similar datasets, we elected to compare sale weights by API internally to detect outliers. Sale weights per year were compared to a mean weight over the sales period, and APIs for which at least one year’s sales weight was more than 10 times greater than the mean were highlighted. The substances are graphed in Figure 12.

**Table 9.** Shortlist of APIs where at least one year’s weight is 10× bigger or smaller than the mean.

API name	Type	Description	Comments
**altrenogest**	sex hormone	veterinary birth control	New formulation (“Altresyn Ceva” authorised in Norway 2018 ( Statens legemiddelverk, 2022)
**asenapine**	antipsychotic	atypical antipsychotic for schizophrenia and bipolar disorder	Sole product (“Syncrest”) deregistered 2017 ( Felleskatalogen, 2022)
**carglumic acid**	metabolic	carbamoyl phosphate synthetase inhibitor for hyperammonaemia	Two products, one of which (“Ucedane”) was first authorised in June 2017 ( Felleskatalogen, 2022)
**cefalotin**	antibacterial	beta-lactam cephalosporin antibiotic	Shortage of cefalotin in Norway recorded 2019 ( Antibiotika.no, 2019)
**cladribine**	antineoplastic	antimetabolite and immunosuppressant for multiple sclerosis and leukaemia	Authorised August 2017 ( Felleskatalogen, 2022)
**cobimetinib**	antineoplastic	mitogen-activated protein kinase inhibitor for melanoma	Authorised November 2015 ( Felleskatalogen, 2022)
**cyclizine**	antiemetic	piperazine antihistamine for nausea relief from motion sickness, vertigo	Cause of change unknown
**dacarbazine**	antineoplastic	alkylating agent for skin cancer and lymphoma	Authorised March 2017 ( Felleskatalogen, 2022)
**dasabuvir**	antiviral	antiviral used in combination for treatment of hepatitis C	Manufacturer withdrew application for dasabuvir/ ombitasvir/paritaprevir/ritonavir in 2016 ( Nye Metoder, 2016); however, ritonavir is also available alone
**edoxaban**	antithrombotic	Factor Xa inhibitor for clotting reduction for strokes, atrial fibrillation, DVT	Authorised June 2015 ( Felleskatalogen, 2022)
**eluxadoline**	antidiarrheal	treatment for diarrhoea from IBS	Authorised as reimbursable prescription 2017, withdrawn from market 2019 ( Statens legemiddelverk, 2017; Felleskatalogen, 2022)
**fomepizole**	antidote	antidote to methanol and antifreeze poisoning	Cause of change unknown
**gadobenic acid**	diagnostic agent	gadolinium contrast agent used for magnetic resonance imaging	Cause of change unknown
**gadodiamide**	diagnostic agent	gadolinium contrast agent used for magnetic resonance imaging	Deregistered 2018 ( Felleskatalogen, 2022)
**glecaprevir**	antiviral	protease inhibitor used in combination with pibrentasvir for hepatitis C	Glecaprevir/pibrentasvir (“Maviret”) Authorised July 2017 ( Felleskatalogen, 2022)
**ixazomib**	antineoplastic	proteasome inhibitor for multiple myeloma	Authorised November 2016 ( Felleskatalogen, 2022)
**nitrofurantoin**	antibacterial	antibiotic for bladder infections	Shortage recorded from 2018–2021 ( VG, 2019)
**nystatin**	antifungal	topical antifungal	Cause of change unknown
**ombitasvir**	antiviral	antiviral taken with paritaprevir and ritonavir for hepatitis C	See dasabuvir
**osimertinib**	antineoplastic	tyrosine kinase inhibitor for non-small cell lung cancer	Authorised February 2016 ( Felleskatalogen, 2022)
**palbociclib**	antineoplastic	selective cyclin-dependent kinase inhibitor for breast cancer	Authorised November 2016 ( Felleskatalogen, 2022)
**paritaprevir**	antiviral	combination treatment for hepatitis C	See dasabuvir
**pibrentasvir**	antiviral	antiviral used in combination for hepatitis C	See glecaprevir
**prednisone**	steroid	corticosteroid and immunosuppressant for many immune and allergic disorders	Shortage recorded 2019 ( Statens legemiddelverk, 2019)
**safinamide**	dopaminergic	MAO inhibitor for Parkinson's	Authorised February 2015 ( Felleskatalogen, 2022)
**toceranib**	antibacterial	receptor tyrosine kinase inhibitor for canine cancers	Deregistered 2019 ( Felleskatalogen, 2022)
**tofacitinib**	immunosuppressant	treatment for arthritis, ulcerative colitis	Authorised March 2017 ( Felleskatalogen, 2022)
**velpatasvir**	antiviral	NS5A inhibitor for hepatitis C	Sofosbuvir/velpatasvir (“Epclusa”), Sofosbuvir/ velpatasvir/voxilaprevir (“Vosevi”) authorised July 2016 ( Felleskatalogen, 2022)
**venetoclax**	antineoplastic	treatment for leukaemia	Authorised December 2016 ( Felleskatalogen, 2022)
**vinflunine**	antineoplastic	alkaloid derivative for bladder cancer	Cause of change unknown
**voxilaprevir**	antiviral	protease inhibitor for hepatitis C	See velpatasvir

API, Active Pharmaceutical Ingredient; DVT, deep vein thrombosis; IBS, irritable bowel syndrome; MAO, monoamine oxidase; NS5A, nonstructural protein 5A.

 This shortlist covered two APIs with exclusively veterinary use (altrenogest and toceranib) and 29 exclusively human APIs. All of the APIs were available exclusively via prescription, except for cyclizine. Registration and deregistration dates were checked, across the APIs, to determine if changes in consumption could be explained by regulatory status. As products, and therefore product API content tend to remain consistent over the 2016–19 period, the above changes are expected to represent actual changes in consumption. However, it was considered prudent to check medical and pharmacy literature for possible explanations, nevertheless ( Table 9).

Stark changes largely corresponded with recorded changes in marketing authorisation (23 substances, 74.1%). Use in some APIs appears to result from shortages in supply (three, 12.5%), while the remaining five (16.1%) were not immediately explicable. These latter substances were then re-checked in source data, no errors were found between years. In three cases, where 2018 sales weights were available from both our and Felleskatalogen data (osimertinib, gadobenic acid and edoxaban), both predictions were in close agreement (<10% difference between values). Beyond the (de)registrations and supply issues listed above, changes in use may be driven by public advertising campaigns, medical lobbying, or relevant press stories.

## Ethics and consent

Ethical approval and consent were not required.

## Data Availability

Open Science Framework: Pharmaceutical pollution: Prediction of environmental concentrations from national wholesales data. https://doi.org/10.17605/OSF.IO/Y74FW ( Welch *et al*., 2022). The following files were used in the creation of this work. All foreground data is included, as is background data publicly available or created by the authors. A published summary of NIPH wholesale data can be found at https://www.fhi.no/en/publ/2021/drug-consumption-in-norway-2016-2020/; in addition to the contact details of relevant NIPH personnel. Data are available under the terms of the Creative Commons Attribution 4.0 International license (CC-BY 4.0).
