# Supplementary material for: Pharmaceutical pollution: Prediction of environmental concentrations from national wholesales data
Source: Open Res Eur. 2022 Sep 15;2:71. Originally published 2022 Jun 1. [Version 2] doi: 10.12688/openreseurope.14129.2 (PMC10445819; doi:10.12688/openreseurope.14129.2)
Supplement: Peer review history

# Peer review: Reviewer response for version 2

**DOI:** 10.21956/openreseurope.16279.r30494

**Published:** 2023-02-13

**Authors:** Rosa Maria Marce

**Affiliations:** 1 Department of Analytical Chemistry and Organic Chemistry, Faculty of Chemistry, Campus Sescelades, Universitat Rovira i Virgili, Tarragona, Spain

The article describes how the Predicted Environmental Concentration (PEC) of the active pharmaceutical ingredient (API) can be estimated from the whole sales data of pharmaceuticals products. The study includes almost 900 APIs used in human and veterinary medicine and covers the annual sales in Norway during the period 2016-2019.

 The first part, the calculation of sales weights of each APIs per year is well described and the way they calculate it is much more correct than the data obtained in some published studies only based on prescription. As regards the second part, the evaluation of PECs is an issue of increasing interest and after the revisions I considered that it is well presented and clearly discussed.  In my opinion, based on the way manuscript is presented and the valuable information included, the manuscript should be approved.

Are sufficient details of methods and materials provided to allow replication by others?

Yes

Is the rationale for creating the dataset(s) clearly described?

Yes

Are the datasets clearly presented in a useable and accessible format?

Yes

Are the protocols appropriate and is the work technically sound?

Yes

Reviewer Expertise:

Occurence of pharmaceuticals in the enviroment. Risk assessment of chemicals.

I confirm that I have read this submission and believe that I have an appropriate level of expertise to confirm that it is of an acceptable scientific standard.

# Peer review: Reviewer response for version 2

**DOI:** 10.21956/openreseurope.16279.r30101

**Published:** 2022-09-26

**Authors:** Gerd Maack

**Affiliations:** 1 German Environment Agency (UBA), Dessau-Roßlau, Germany

I have no further comments to the revised version of the manuscript.

Are sufficient details of methods and materials provided to allow replication by others?

Yes

Is the rationale for creating the dataset(s) clearly described?

Yes

Are the datasets clearly presented in a useable and accessible format?

Partly

Are the protocols appropriate and is the work technically sound?

Yes

Reviewer Expertise:

Environmental Risk Assessment of Pharmaceuticals. Authorization of Pharmaceutical Products. Endocrine Disruption

I confirm that I have read this submission and believe that I have an appropriate level of expertise to confirm that it is of an acceptable scientific standard.

# Peer review: Reviewer response for version 1

**DOI:** 10.21956/openreseurope.15234.r29467

**Published:** 2022-06-28

**Authors:** Ad M. J. Ragas, Caterina Zillien

**Affiliations:** 1 Department of Environmental Science, Radboud Institute for Biological and Environmental Sciences, Radboud University, Nijmegen, The Netherlands

Summary of the article

 Welch *et al*. 2022 describes a methodology to convert national wholesales data of almost 900 APIs used in human and veterinary medicine into a dataset that can be used to estimate environmental exposure data. The resulting dataset covers annual wholesales from Norway for the period 2016 – 2019 and provides a comprehensive overview of API sales for an entire country. The different sources to obtain the data and their scopes and limitations are well described and compared to each other.

General impression

 This is a nice data note on a highly relevant topic. The note explains how whole sales data of pharmaceutical products can be used to predict environmental concentration of Active Pharmaceutical Ingredients (APIs). The first part of this exercise is interesting, i.e. the step from wholesales data of pharmaceutical products to the amount of API that is sold. This is also the part that is being validated; or at least comparisons are made with other studies, adding to the trustworthiness of the method. The second part of the method, i.e. the prediction of the PEC (and any references made to prioritization and PNECs) are less convincing. The PEC is estimated in a very rudimentary way; hardly the state-of-the-art. The predictions are also not explicitly compared to measured values and thus not validated. We suggest removing this part from the manuscript.

Points of concern:

- The authors correctly mention that some APIs are salts. Where the PNEC is typically reported as the amount (i.e., weight) of the active ion, products typically report the weight of the salt. This can result in errors. The authors mention this, but they do not explicitly state how they dealt with this issue. Do the API weights that they report refer to the salt or to the active ion? And how did they deal with different salts that have the same active ion? The authors should be more explicit about their implicit assumptions on this point.
- To derive the PEC, no API-specific excretion was considered. This results in the overestimation of PEC but is not mentioned explicitly in section ‘Predicted Environmental Concentrations’. This is one of the reasons, we suggest removing the whole section on PECs and to focus on the estimation on the API sales weights.
- The section on Potential Applications is rather speculative. It does not belong in a methods section. We’re not familiar with the formal structure of a data note, but it seems more appropriate to put this type of argument in a reflection/discussion section.
- For your international audience, it would be great if the titles of the datasets on the repository were in English.
- Not all data (i.e., the NIPH wholesales data) used in the data note seem to be publicly accessible. As such, it is difficult to reproduce the results. We don't find this a huge problem, but we're not sure whether this is in line with the publication policy of the journal.

Specific comments

 P1: more prominent groups -> please specify;

 P1: We doubt whether all readers will know the difference between market-based and sales-based assessments;

 P1: Is ecotoxicological-exempt the same as data deficient?

 P2: Human biology -> what about the veterinary pharmaceuticals?

 P2: but doing so everywhere -> doing what everywhere? I assume measuring, but this is not explicitly stated;

 P2: Somewhere you should explain in a bit more detail what the difference is between wholesales data and prescription data. Figure 2 nicely captures this.

 P6:  The main data tables are shown in Figure 4 -> the tables in Figure 4 have different names than the main data tables listed in the text.

 Confusing.

 P6: the associated API names associated were…

 P8: validating sales data is definitely not enough to “quality-assure PECs”. Please remove or reformulate.

 P9: Please add a more explanatory caption. What does “non-masses”, “real masses” and “returns” refer to?

 P9: The Norwegian Prescription Database (NorPD), the Norwegian Prescription Database…

 P11:  Numbers in text are reported in a lot of detail. I suggest using a scientific notation to avoid the suggestion of too much accuracy.

 P12: Remove Figure 7b. It adds little to no new information.

 P12: More dated -> do you mean more recent?

 P14/15:The legend of Figures 9-11 is not particularly clear. Numbers are also difficult to compare. Can you find a different, more transparent way of presenting these results?

 P16: Table 9: Nice example of how this data can be used to detect interesting trends (and/or mistakes).

 P17: Some of the names of the data files could be a bit more user-friendly so that the reader immediately understands the content.

Are sufficient details of methods and materials provided to allow replication by others?

No

Is the rationale for creating the dataset(s) clearly described?

Yes

Are the datasets clearly presented in a useable and accessible format?

Yes

Are the protocols appropriate and is the work technically sound?

Partly

Reviewer Expertise:

Human and ecological risk assessment of chemicals, particularly pharmaceuticals.

We confirm that we have read this submission and believe that we have an appropriate level of expertise to confirm that it is of an acceptable scientific standard, however we have significant reservations, as outlined above.

# Response

**Published:** 2022-08-21

**Authors:** Sam Welch

**Affiliations:** Norwegian institute for water research, Norway

Thank you for your quick and comprehensive feedback on our paper. I’ve revised the paper in response to a number of your suggestions, and I’ll attempt to respond to them all below.

**General impression**

**This is a nice data note on a highly relevant topic. The note explains how whole sales data of pharmaceutical products can be used to predict environmental concentration of Active Pharmaceutical Ingredients (APIs). The first part of this exercise is interesting, i.e. the step from wholesales data of pharmaceutical products to the amount of API that is sold. This is also the part that is being validated; or at least comparisons are made with other studies, adding to the trustworthiness of the method. The second part of the method, i.e. the prediction of the PEC (and any references made to prioritization and PNECs) are less convincing. The PEC is estimated in a very rudimentary way; hardly the state-of-the-art. The predictions are also not explicitly compared to measured values and thus not validated. We suggest removing this part from the manuscript.**

*The PEC is indeed calculated in a rudimentary way; unfortunately, with 800+ APIs over four years and limited time this seemed like the best compromise to make the data publicly available. I would also note that more precise modelling tools, such as Oldenkamp et al.’s ePiE are not yet set up for Norway. Our approach is crude, but we’re limited by the tools we have available, while removing the PECs entirely would make this data paper no longer an ecotoxicological resource. I’ve expanded the discussion in the introduction more to cover these questions, but I believe too much discussion would, again, be out of the scope of a data note.*

**Points of concern:** **The authors correctly mention that some APIs are salts. Where the PNEC is typically reported as the amount (i.e., weight) of the active ion, products typically report the weight of the salt. This can result in errors. The authors mention this, but they do not explicitly state how they dealt with this issue. Do the API weights that they report refer to the salt or to the active ion? And how did they deal with different salts that have the same active ion? The authors should be more explicit about their implicit assumptions on this point.**

*I’ve attempted to clarify this in the methods section, but in essence: when clear data on the salt form of an API was available, we factored it into our concentration. When it wasn’t, we assumed the full weight corresponded to the active ion.*

**To derive the PEC, no API-specific excretion was considered. This results in the overestimation of PEC but is not mentioned explicitly in section ‘Predicted Environmental Concentrations’. This is one of the reasons, we suggest removing the whole section on PECs and to focus on the estimation on the API sales weights.**

*Acquiring or developing API-specific excretion factors for 800+ APIs was beyond the scope of this paper. This does potentially lead to overestimates of risk, especially for well-metabolised APIs, but as it’s also possible for metabolites to be more toxic, or transformed back into toxic products in the environment, we believe modelling excretion as negligible provides a safest worst-case approach. I’ve added a summary of this to the section of Predicted Environmental Concentrations*.

**The section on Potential Applications is rather speculative. It does not belong in a methods section. We’re not familiar with the formal structure of a data note, but it seems more appropriate to put this type of argument in a reflection/discussion section.**

*This is a reasonable point. I’ve removed the section to keep the paper streamlined – it was an inclusion from an earlier version of the paper and wasn’t described in the data note guidelines. We’ll cover applications further in an upcoming paper, and they’re also mentioned in the Deliverable D6.2 linked in the introduction.*

**For your international audience, it would be great if the titles of the datasets on the repository were in English.** I’ve updated the names of all data sets to English. **Not all data (i.e., the NIPH wholesales data) used in the data note seem to be publicly accessible. As such, it is difficult to reproduce the results. We don't find this a huge problem, but we're not sure whether this is in line with the publication policy of the journal.**

*The author's guidelines state: "Data notes must describe research data generated and owned by the authors." We’ve published all the foreground data, generated by the project (Figure 3f & g), some publicly available data, but no background data owned by other parties/under commercial confidentiality. I’ve updated the Data availability section to make it more explicit which data we are and aren’t able to publish.*

**Specific comments** *Responses to the following comments have been limited to save space, but they have all been addressed.*

**P14/15:The legend of Figures 9-11 is not particularly clear. Numbers are also difficult to compare. Can you find a different, more transparent way of presenting these results?**

 I’ve spent some time considering alternative ways to display the data, but ultimately, I feel these graphs allow comparison between multiple datasets without creating a false conception of closeness. Sales in DDD/1000/day and kg are not directly comparable, especially across different combination ATC codes, but trends map to each other, and sales are plausible taking into account growth in consumption since 2005. **Are sufficient details of methods and materials provided to allow replication by others? – **No In our view, the question of replication (of results) by others is not strictly relevant for a data note. The "methods" are provided as R codes. However, the "materials" would correspond to background data owned by others (NIPH) which cannot be published here. Therefore, the "results" (the foreground data published here) cannot be replicated by others.

# Peer review: Reviewer response for version 1

**DOI:** 10.21956/openreseurope.15234.r29470

**Published:** 2022-06-22

**Authors:** Gerd Maack

**Affiliations:** 1 German Environment Agency (UBA), Dessau-Roßlau, Germany

The data for this manuscript is part of a larger project and utilize the unique Norwegian Wholesale Statistic database.

 However, the text is quite difficult to read, as it misses an overall red line, especially for readers not involved in the project and those who did not read the project report.

 One example of this is the data evaluation. For me, it is not clear why the author chose the data and publications they compared the results of this project to. Grung *et al. *(2005) and the Felleskatalogen data are very likely not known to anyone outside of Norway. Here a better explanation would have been needed.

 Finally, all the effort of building the database and extracting the data should end in using the database and producing results. The results, presented here are, in my opinion, not really representative.  The criteria chosen, where at least one year’s weight is 10x different than the mean, is at minimum unique. I would have expected a bigger evaluation and more results. What is with e.g. the Top Ten of the highest consumption in Norway? What is with the usual suspects like Metformin, Ibuprofen, Diclofenac, etc….? Or with substances which are known to display an environmental risk?

 I, therefore, find this manuscript is not really suitable for indexing.

 Some detailed comments.

1. Grung (2005) In Figure 9 -11 Grung (2005) is cited, which is not in the references and also not mentioned in the text.
2. Dilution factor - In table 7 the PECsw equation default variables, used in the EMA guideline, are described. In the respective text, it is mentioned that the default dilution factor of 10 is quite conservative. This might be correct for Norway with the unique combination of large fjords and a small overall population. However, the water exchange in some fjords might be quite low, due to the length and the shape and therefore hardly any tidal currents and already in the Olso region, it is probably a different matter. Especially in other parts of Europe, this is clearly not correct. See therefore the public press of the effluent concentration in British rivers and e.g. Link *et al*. ^1^ for rivers in Germany.
3. Independent of the above, an exposure scenario, where the effluent is discharged directly into the marine environment is not included in the EMA guideline.
4. Comparison with prescription data - Individual active ingredients are sold both as OTC-products and as prescription products, depending on form and strength. This is missing in the discussion on the gap between prescription and sales data.
5. Checking for extreme changes - Reasons for differences can also be an adverb campaign for new generics (increasing consumption) or a similar adverb campaign of a competitor (decreasing consumption)

Are sufficient details of methods and materials provided to allow replication by others?

Yes

Is the rationale for creating the dataset(s) clearly described?

Yes

Are the datasets clearly presented in a useable and accessible format?

Partly

Are the protocols appropriate and is the work technically sound?

Yes

Reviewer Expertise:

Environmental Risk Assessment of Pharmaceuticals. Authorization of Pharmaceutical Products. Endocrine Disruption

I confirm that I have read this submission and believe that I have an appropriate level of expertise to confirm that it is of an acceptable scientific standard, however I have significant reservations, as outlined above.

## References

[1] : Comparison of dilution factors for German wastewater treatment plant effluents in receiving streams to the fixed dilution factor from chemical risk assessment. *Sci Total Environ* .2017;598: 10.1016/j.scitotenv.2017.04.180 805-813 10.1016/j.scitotenv.2017.04.180 28458197

# Response

**Published:** 2022-08-21

**Authors:** Sam Welch

**Affiliations:** Norwegian institute for water research, Norway

Thank you for your quick and comprehensive feedback on our paper. I’ve revised the paper in response to a number of your suggestions, and I’ll attempt to respond to them all below. **The data for this manuscript is part of a larger project and utilize the unique Norwegian Wholesale Statistic database.**

**However, the text is quite difficult to read, as it misses an overall red line, especially for readers not involved in the project and those who did not read the project report.** I’ve rewritten part of the abstract and introduction, and I hope our intentions – to calculate PECs from Norwegian drug sales, and publish them – are clearer now.

**One example of this is the data evaluation. For me, it is not clear why the author chose the data and publications they compared the results of this project to. Grung *et al. *(2005) and the Felleskatalogen data are very likely not known to anyone outside of Norway. Here a better explanation would have been needed.** Pharmaceuticals sales data is not generally publicly available, in Norway or elsewhere, and both predicted and measured environmental concentration data for Norway are similarly scarce, compared with better-studied nations such as Germany. Grung et al. (2008) was the only previously published ecotoxicological exercise conducted with the Norwegian Wholesale Database, so we wanted to ensure that the sales weights we calculated were consistent with expected growth in consumption since 2008. Likewise, Felleskatalogen represents the only public source of PECs for APIs in Norway, but as far as we know their results are not archived year-on-year and are not transparent. As Felleskatalogen PECs are predicted using sales data from a private market research firm, this represented one of the few options we had to check for agreement between two sources of the same data. I’ve attempted to clarify these points in the section Data evaluation.

**Finally, all the effort of building the database and extracting the data should end in using the database and producing results. The results, presented here are, in my opinion, not really representative.  ** ORE guidelines request that data notes omit analysis and focus on describing the data and its collection/creation, so we believe an analysis would be out of scope. **The criteria chosen, where at least one year’s weight is 10x different than the mean, is at minimum unique. I would have expected a bigger evaluation and more results. What is with e.g. the Top Ten of the highest consumption in Norway? What is with the usual suspects like Metformin, Ibuprofen, Diclofenac, etc….? Or with substances which are known to display an environmental risk?** As above, as a data note more in-depth analysis would be out of scope for the paper. Checking for extreme variation in sales weights was an internal quality-control process for us to assess potential issues in our data, but we elected to include a summary of this covering APIs where considerable changes are present but caused by market factors.

**I, therefore, find this manuscript is not really suitable for indexing.** We hope that our explanations above will prove that the manuscript is suitable for publication in ORE after all, when considering the definition and scope of a Data Note.

**Some detailed comments. **

1. **Grung (2005) In Figure 9 -11 Grung (2005) is cited, which is not in the references and also not mentioned in the text.**

Updated to 2008.

1. **Dilution factor - In table 7 the PECsw equation default variables, used in the EMA guideline, are described. In the respective text, it is mentioned that the default dilution factor of 10 is quite conservative. This might be correct for Norway with the unique combination of large fjords and a small overall population. However, the water exchange in some fjords might be quite low, due to the length and the shape and therefore hardly any tidal currents and already in the Olso region, it is probably a different matter. Especially in other parts of Europe, this is clearly not correct. See therefore the public press of the effluent concentration in British rivers and e.g. Link *et al*.** **^1^** **for rivers in Germany.**

As this study is limited to predicting environmental concentrations in Norway, I believe the comment stands. I’ve found minimal measured or modelled Dilution Factors for Norwegian surface waters, marine or freshwater, which is why we elected to use the default figure of 10. As a side note, fjord-releasing WWTP in Norway typically release effluent from a pipe located low and far from the coast. I’ve added a brief discussion of the choice of DF, including the paper you reference, to the relevant section in Methods. **Independent of the above, an exposure scenario, where the effluent is discharged directly into the marine environment is not included in the EMA guideline.** This is an issue with the EMA guidelines, but not one we had the capacity to address in this work. I’ve added a brief discussion of modelling of saltwater to the section on Predicted Environmental Concentrations.

1. **Comparison with prescription data - Individual active ingredients are sold both as OTC-products and as prescription products, depending on form and strength. This is missing in the discussion on the gap between prescription and sales data.**

I’ve clarified the language around this in Methods: Data sources and management.

1. **Checking for extreme changes - Reasons for differences can also be an adverb campaign for new generics (increasing consumption) or a similar adverb campaign of a competitor (decreasing consumption)**

This is potentially the case, although I doubt it was an important driver compared to the already identified regulatory factors, and I’ve therefor not mentioned it in the test. **Is the rationale for creating the dataset(s) clearly described? - Yes** **Are the protocols appropriate and is the work technically sound? - Yes** **Are sufficient details of methods and materials provided to allow replication by others? - Yes** **Are the datasets clearly presented in a useable and accessible format? - Partly** We’ve attempted to improve the presentation of the published dataset by rendering names in English and with more frequent reference to the data processing pathway depicted in Figure 3.
